# Cytotoxic and Anti-Inflammatory Eunicellin-Based Diterpenoids from the Soft Coral *Cladiella krempfi*

**DOI:** 10.3390/md11030788

**Published:** 2013-03-12

**Authors:** Chi-Jen Tai, Jui-Hsin Su, Chiung-Yao Huang, Ming-Shyan Huang, Zhi-Hong Wen, Chang-Feng Dai, Jyh-Horng Sheu

**Affiliations:** 1 Department of Marine Biotechnology and Resources, National Sun Yat-sen University, Kaohsiung 804, Taiwan; E-Mails: jean801023@hotmail.com (C.-J.T.); betty8575@yahoo.com.tw (C.-Y.H.); wzh@mail.nsysu.edu.tw (Z.-H.W.); 2 National Museum of Marine Biology & Aquarium, Pingtung 944, Taiwan; E-Mail: x2219@nmmba.gov.tw; 3 Graduate Institute of Marine Biotechnology, National Dong Hwa University, Pingtung 944, Taiwan; 4 Division of Pulmonary and Critical Care Medicine, Department of Internal Medicine, Kaohsiung Medical University Hospital, Kaohsiung 807, Taiwan; E-Mail: shyang@cc.kmu.edu.tw; 5 Center of Excellence for Environmental Medicine, Kaohsiung Medical University, Kaohsiung 807, Taiwan; 6 Institute of Oceanography, National Taiwan University, Taipei 112, Taiwan; E-Mail: corallab@ntu.edu.tw; 7 Division of Marine Biotechnology, Asia-Pacific Ocean Research Center, National Sun Yat-sen University, Kaohsiung 804, Taiwan

**Keywords:** eunicellin-based diterpenoids, *Cladiella krempfi*, cytotoxicity, anti-inflammatory agents

## Abstract

Five new eunicellin-based diterpenoids, krempfielins E–I (**1**–**5**) and seven known compounds (**6**–**12**) were isolated from the organic extract of a Taiwanese soft coral *Cladiella krempfi*. The structures of the new metabolites were elucidated on the basis of extensive spectroscopic analysis. Metabolites **5**, **6**, **10** and **12** were shown to exhibit cytotoxicity against a limited panel of cancer cell lines. Furthermore, compounds **6** and **10** could potently inhibit the accumulation of the pro-inflammatory iNOS protein, and **6** and **12** could significantly reduce the expression of COX-2 protein in LPS-stimulated RAW264.7 macrophage cells.

## 1. Introduction

**Chart 1:**
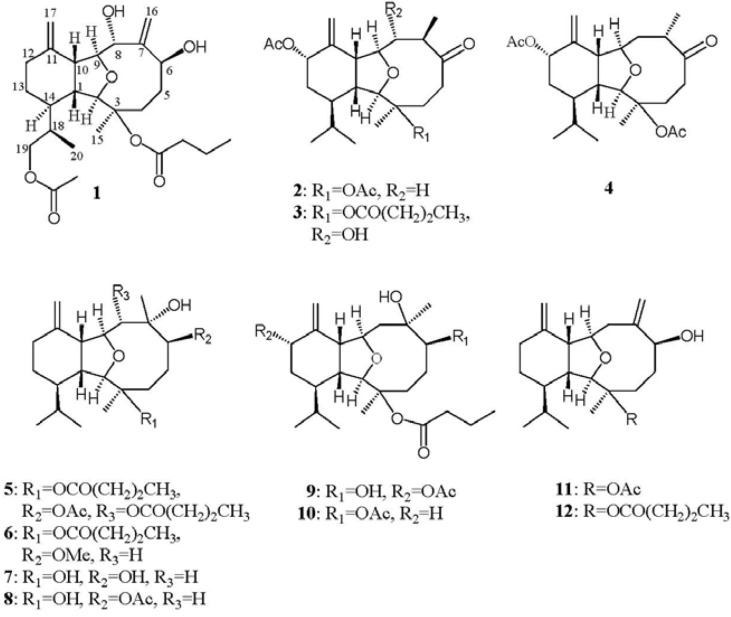
Structures of metabolites **1**–**12**.

Previous studies showed that many eunicellin-based diterpenes discovered from soft corals exhibited cytotoxic and anti-inflammatory activities [1,2,3,4,5,6,7,8,9,10,11,12,13,14]. The soft coral *Cladiella krempfi* has been found to generate several types of metabolites including eunicellin-type diterpenoids [15] and pregnane-type steroids [16,17,18]. Our previous chemical investigation of *C. krempfi* had led to the isolation of four new eunicellin-based diterpenoids, krempfielins A–D [19]. In this paper, we further report the isolation of five new eunicellin-based diterpenoids, krempfielins E–I (**1**–**5**) (Chart 1) and known compounds 6-methyl ether of litophynol B (**6**) [13], sclerophytin A (**7**) [20], sclerophytin B (**8**) [20], litophynin I monoacetate (**9**) [21], 6-acetoxy litophynin E (**10**) [22], (1R*, 2R*, 3R*, 6S*, 9R*, 10R*, 14R*)-3-acetoxycladiell-7(16),11(17)-dien-6-ol (**11**) [23] and litophynin F (**12**) [24]. The structures of metabolites **1**–**12** were characterized by extensive spectroscopic analysis. Cytotoxicity of all compounds against five human tumor cell lines, lung adenocarcinoma (A549 and H1299), breast carcinoma (BT483), liver carcinoma (HepG2), oral cancer (SAS) and one human lung bronchial cell (BEAS2B) lines was studied. The ability of them to inhibit the up-regulation of pro-inflammatory iNOS (inducible nitric oxide synthase) and COX-2 (cyclooxygenase-2) proteins in LPS (lipopolysaccharide)-stimulated RAW264.7 macrophage cells was also evaluated. The results showed that compounds **5**, **6**, **10** and **12**, in particular **10**, are cytotoxic towards the above cancer cell’s compounds. Except **2**, **4** and **11**, these compounds were found to significantly reduce the levels of iNOS protein; among them, **6** and **10** are most active. Furthermore, **6** and **12** also could significantly reduce the expression of COX-2 protein.

## 2. Results and Discussion

**Figure 1:**
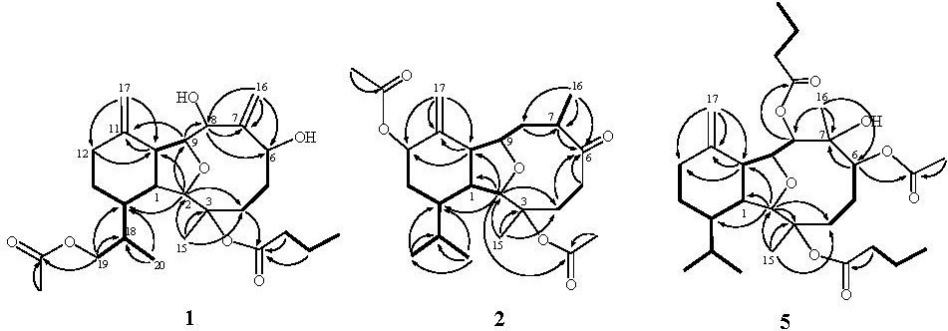
Selected ^1^H–^1^H COSY (▬) and HMBC (→) correlations of **1**, **2** and **5**.

The new metabolite krempfielin E (**1**) showed the molecular ion peak [M + Na]^+^ at *m*/*z* 487.2669 in the HRESIMS and established a molecular formula of C_26_H_40_O_7_, implying seven degrees of unsaturation. The IR absorptions bands at n_max_ 3421 and 1732 cm^−1^ revealed the presence of hydroxy and ester carbonyl functionalities. The ^13^C NMR spectrum measured in CDCl_3_ showed signals of twenty-six carbons (Table 1) which were assigned by the assistance of the DEPT spectrum to four methyls (including one acetate methyl δ_C_ 21.0), seven sp^3^ methylenes, two sp^2^ methylenes, eight sp^3^ methines (including four oxymethines), one sp^3^ and four sp^2^ quaternary carbons (including two ester carbonyls).The NMR spectroscopic data of **1** (Table 1, Table 2) showed the presence of two 1,1-disubstituted double bonds (δ_C_ 118.1 CH_2_, 112.0 CH_2_, 152.2 C, and 145.2 C; δ_H_ 5.51 s, 5.22 s, 4.81 s, and 4.65 s). Two ester carbonyls (δ_C_ 172.5 and 171.2) were assigned from the ^13^C NMR spectrum and their signals were correlated with the methylene protons (δ_H_ 2.10, 2H, m) of an *n*-butyrate and protons of an acetate methyl (δ_H_ 2.07 s, 3H), respectively. Therefore, the remaining three degrees of unsaturation identified **1** as a tricyclic molecule. The ^1^H–^1^H COSY and HMBC correlations (Figure 1) were further used for establishing the molecular skeleton of **1**. The COSY experiment assigned three isolated consecutive proton spin systems. Above evidences and the analysis of HMBC spectrum (Figure 1) suggested that **1** is an eunicellin-based diterpenoid. Furthermore, the acetoxy group attaching at C-19 was confirmed by the HMBC correlations from oxymethylene [δ_H_ 3.94 (H_2_-19)] and acetate methyl protons (δ_H_ 2.07) to the ester carbonyl carbon appearing at δ 171.2 (C). Thus, the remaining one *n*-butyryloxy group had to be positioned at C-3, an oxygen-bearing quaternary carbon resonating at δ 84.4 ppm. On the basis of above analysis, the planar structure of **1** was established. 

**Table 1:** ^13^C NMR Data for Compounds **1**–**5**.

C	1 ^a^	2 ^a^	3 ^b^	4 ^b^	5 ^a^
1	43.3, CH ^c^	45.8, CH	44.7, CH	44.9, CH	46.6, CH
2	91.4, CH	90.6, CH	90.9, CH	90.8, CH	92.6, CH
3	84.4, C	84.7, C	84.2, C	84.6, C	85.6, C
4	28.4, CH_2_	33.2, CH_2_	33.2, CH_2_	32.6, CH_2_	35.8, CH_2_
5	35.3, CH_2_	30.2, CH_2_	29.7, CH_2_	38.3, CH_2_	28.5, CH_2_
6	66.9, CH	214.8, C	217.0, C	213.4, C	82.3, CH
7	152.2, C	48.3, CH	40.7, CH	40.6, CH	78.0, C
8	77.3, CH	42.4, CH_2_	78.5, CH	37.7, CH_2_	78.5, CH
9	83.7, CH	81.9, CH	85.5, CH	78.9, CH	78.3, CH
10	47.9, CH	52.1, CH	47.5, CH	49.4, CH	52.0, CH
11	145.2, C	142.1, C	141.0, C	141.6, C	148.5, C
12	31.3, CH_2_	73.1, CH	72.8, CH	73.0, CH	31.6, CH_2_
13	25.7, CH_2_	28.5, CH_2_	28.9, CH_2_	28.7, CH_2_	25.5, CH_2_
14	39.1, CH	35.3, CH	35.6, CH	35.7, CH	44.0, CH
15	22.2, CH_3_	22.3, CH_3_	22.6, CH_3_	22.6, CH_3_	22.8, CH_3_
16	118.1, CH_2_	17.8, CH_3_	14.1, CH_3_	15.4, CH_3_	18.5, CH_3_
17	112.0, CH_2_	118.1, CH_2_	119.5, CH_2_	118.7, CH_2_	109.2, CH_2_
18	32.6, CH	28.0, CH	27.2, CH	27.5, CH	29.0, CH
19	67.5, CH_2_	21.5, CH_3_	21.6, CH_3_	21.5, CH_3_	21.9, CH_3_
20	10.6, CH_3_	16.6, CH_3_	14.9, CH_3_	14.9, CH_3_	15.4, CH_3_
3-*n*-butyrate	172.5, C		172.5, C		173.1, C
	37.4, CH_2_		37.3, CH_2_		36.5, CH_2_
	18.5, CH_2_		18.4, CH_2_		18.4, CH_2_
	13.6, CH_3_		13.7, CH_3_		13.8, CH_3_
3-OAc		169.6, C		169.7, C	
		22.4, CH_3_		22.3, CH_3_	
6-OAc					171.6, C
					21.4, CH_3_
8-*n*-butyrate					173.3, C
					36.7, CH_2_
					18.2, CH_2_
					13.5, CH_3_
12-OAc		170.2, C	170.1, C	170.2, C	
		21.6, CH_3_	21.5, CH_3_	21.4, CH_3_	
19-OAc	171.2, C				
	21.0, CH_3_				

^a^ Spectra recorded at 100 MHz in CDCl_3_. ^b^ Spectra recorded at 125 MHz in CDCl_3_. ^c^ Deduced from DEPT.

**Table 2:** ^1^H NMR Data for Compounds **1**–**5**.

H	1 ^a^	2 ^a^	3 ^b^	4 ^b^	5 ^a^
1	2.25 m	2.25 dd	2.28 m	2.29 m	2.17 m
		(12.4, 7.2)			
2	3.71 br s	3.67 br s	3.81 br s	3.73 br s	3.63 br s
4	1.64 m 2.25 m	2.03 m 2.75 dd (14.4, 8.8)	2.23 m 2.46 m	2.19 m 2.52 dd (5.5, 2.0)	1.96 m 2.65 m
5	1.74 m 2.19 m	1.91 m 2.48 dd (13.2, 11.2)	1.25 m	2.25 m 2.43 t (10.5)	1.56 m
6	4.73 dd				5.71 d (5.6)
	(11.2, 4.0) ^c^				
7		2.58 m	2.32 m	2.68 m	
8	4.18 d (3.2)	1.88 m	3.77 d (8.5)	1.87 t (5.0)	5.27 d (9.2)
		2.00 m		2.24 m	
9	4.19 d (6.8)	4.12 ddd	4.43 d (11.0)	4.23 dt	4.11 t (8.8)
		(11.6, 8.8, 4.4)		(10.0, 5.0)	
10	2.87 dd	3.12 t (7.6)	2.91 t (7.5)	3.07 td	3.39 t (7.2)
	(10.8, 8.0)			(10.0, 1.5)	
12	2.10 m	5.49 t (2.8)	5.52 br s	5.49 t (2.5)	2.03 m
	2.27 m				2.23 m
13	1.11 m 1.70 m	1.26 m 1.92 m	1.32 m 1.97 dt (14.5, 3.0)	1.29 m 1.94 dt (14.5, 3.0)	1.08 m 1.76 m
14	1.55 m	1.71 m	1.76 m	1.71 m	1.23 m
15	1.63 s	1.40 s	1.49 s	1.48 s	1.38 s
16	5.22 s	1.06 d (7.2)	1.26 d (6.5)	1.07 d (7.0)	1.08 s
	5.51 s				
17	4.65 s	4.99 d (1.6)	4.96 s	4.97 s	4.51 s
	4.81 s	5.91 d (1.6)	5.27 s	5.28 s	4.64 s
18	2.08 m	1.76 m	1.84 m	1.81 m	1.69 m
19	3.94 t (6.4)	0.91 d (6.8)	0.96 d (7.0)	0.93 d (6.5)	0.96 d (6.8)
20	0.80 s	0.75 d (6.8)	0.78 d (7.0)	0.77 d (6.5)	0.77 d (6.8)
3-*n*-butyrate	2.10 m		2.28 m		2.25 m
	1.57 m		1.68 m		1.68 m
	0.92 t (7.2)		0.99 t (7.5)		0.98 t (7.6)
3-OAc		2.18 s		2.14 s	
6-OAc					2.09 s
8-*n*-butyrate					2.55 m
					1.61 m
					0.96 t (7.2)
12-OAc		2.00 s	2.03 s	1.98 s	
19-OAc	2.07 s				

^a^ Spectra recorded at 400 MHz in CDCl_3_. ^b^ Spectra recorded at 500 MHz in CDCl_3_. ^c^ *J* values (Hz) in parentheses.

**Figure 2:**
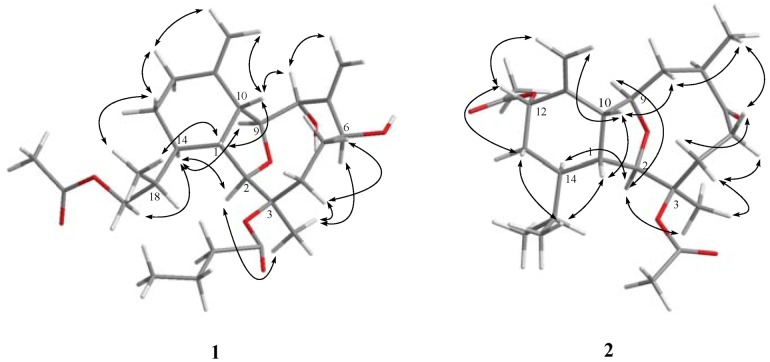
Key NOESY correlations for **1** and **2**.

The relative structure of **1** was elucidated by the analysis of NOE correlations, as shown in Figure 2. The observation of the NOE correlations of H-1 with H-10 and H_3_-20 suggested that these protons had the same orientation and were assumed to be β-oriented. The NOE interactions found between the oxymethine proton H-8 and H-10 assigned the α-orientation of the hydroxy group. Furthermore, the NOE correlations of H-2 with both H-14 and H_3_-15; H-14 with both H-9 and H_2_-19; and H_3_-15 with H-6, suggested that H-2, H-6, H-9, H-14, and H_3_-15 are α-oriented. Furthermore, the configuration of C-18 was to be R* on the basis of NOE correlations of H-1/H_3_-20 and H-14/H_2_-19. The relative configuration of **1** was thus established. Comparison of the ^1^H and ^13^C NMR spectroscopic data of **1** with those of its 19-deacetoxyl derivative, litophynol A [21], further confirmed the structure of **1**.

**Figure 3:**
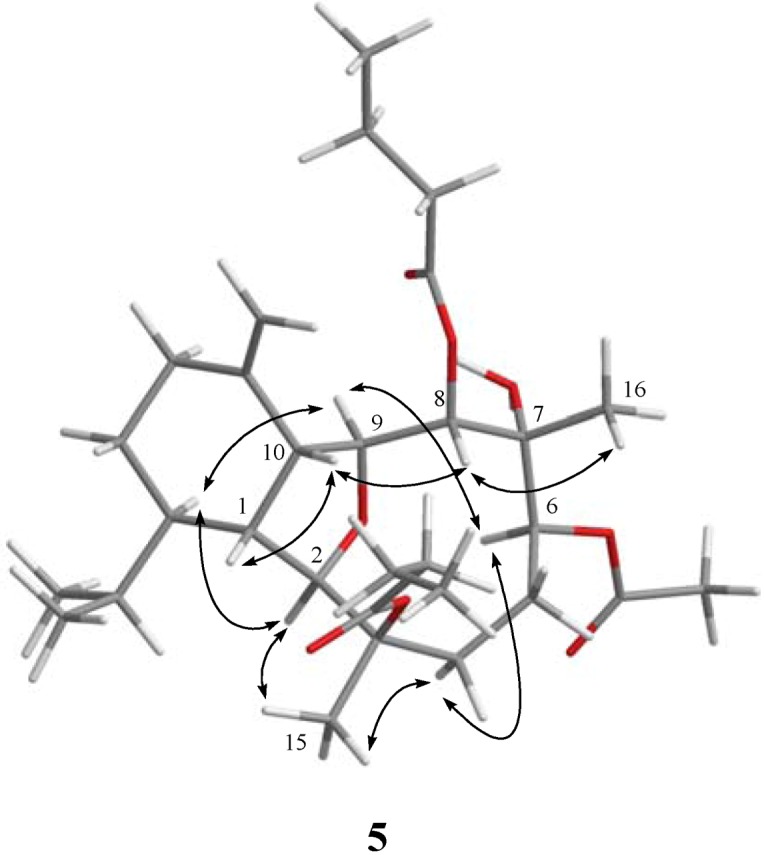
Key NOESY correlations for **5**.

Krempfielin F (**2**) was found to have the molecular formula C_24_H_36_O_6_ and seven degrees of unsaturation, as indicated from the HRESIMS (*m*/*z* 443.2408 [M + Na]^+^). The ^13^C NMR spectrum of **2** showed signals of twenty-four carbons (Table 1), which were characterized by the DEPT spectrum as six methyls, five methylenes (including one sp^2^ methylene), eight methines (including three oxygenated carbons), and five quaternary carbons (including one ketone carbonyl, two ester carbonyls, and one sp^2^ quaternary carbon of an olefinic group). The presence of two acetoxy groups was indicated by the ^1^H NMR signals (Table 2) the ^1^H NMR signals at δ_H_ 2.18 (s, 3H) and 2.00 (s, 3H), and the ^13^C NMR signals at δ_C_ 22.4 (CH_3_), 21.6 (CH_3_), 169.6 (C), and 170.2 (C). The remaining three degrees of unsaturation again identified **2** as a tricyclic diterpenoid. The molecular framework was established by ^1^H–^1^H COSY and HMBC experiments (Figure 1). The stereochemistry of compound **2** was also determined by the NOESY spectrum (Figure 3), which exhibited NOE correlations of H-1 and with H-10 and H_3_-20, H-13β (δ_H_ 1.26) and with H-12 and H_3_-20, H-8β (δ_H_ 1.88) with H-5β (δ_H_ 2.48), H-10 and H_3_-16, H-5α with H-4α and H_3_-15, and H_3_-16 with both H-8β (δ_H_ 1.88) and H-5β (δ_H_ 2.48), establishing the β-orientation of H-12 and H_3_-16. On the basis of these results and other observed NOE correlations (Figure 2), the structure of metabolite **2** was determined.

The HRESIMS (*m*/*z* 487.2675 [M + Na]^+^) of **3** established the molecular formula of C_26_H_40_O_7_. Comparison of the NMR data of **3** with those of **2** revealed the replacement of one acetoxy group (δ_H_ 2.18, 3H, s; δ_C_ 169.6, C and 22.4, CH_3_) in **2** by an *n*-butyryloxy group in **3** (δ_H_ 0.99, 3H, t, *J* = 7.5 Hz; 1.68, 2H, m; 2.28, 2H, m; and δ_C_ 172.5, C; 37.3, CH_2_; 18.4, CH_2_ and 13.7, CH_3_), and an additional hydroxy group substituted at C-8 of **3** that downfielded H-8 to δ_H_ 3.77 and C-8 to δ_C_ 78.5 ppm. The placement of the *n*-butyryloxy group at C-3 was confirmed by the HMBC experiment which showed a correlation between H-2 and the carbonyl carbon (δ_C_ 172.5 C) of this *n*-butyryloxy group. The NOE correlations of **3** also showed that the stereochemistry of this metabolite is identical with that of **2** excepted for the presence of the α-oriented hydroxy group at C-8. 

The HRESIMS of krempfielin H (**4**) exhibited a [M + Na]^+^ ion peak at *m*/*z* 443.2408, appropriate for a molecular formula of C_24_H_36_O_6_. By analysis of 2D NMR spectra, including ^1^H–^1^H COSY, HMQC, and HMBC, compound **4** was shown to possess the same molecular framework as that of **2**. Furthermore, it was found that the NMR data of **4** were very similar to those of **2** (Table 1, Table 2), suggesting that **4** might be an isomer of **2**. From NOESY spectrum, it was found that the β-oriented H-10 showed NOE interactions with both H-7 and H-8β (δ_H_ 1.87), while H-8β showed NOE interactions with H-7, indicating the β-orientation of H-7. This inferred the α-orientation of methyl substituent at C-7. Further analysis of other NOE interactions revealed that **4** possessed the same relative configuration sat C-1, C-2, C-3, C-9, C-10, C-12 and C-14 as those of **2**. Therefore, **4** was found to be the C-7 epimer of **2**.

The related metabolite, krempfielin I (**5**), had a molecular formula of C_30_H_48_O_8_ as indicated by the HRESIMS (*m/z* 559.3243, [M + Na]^+^) and NMR data (Table 1, Table 2). The ^13^C NMR spectrum of **5** revealed the appearance of three ester carbonyls (δ_C_ 173.3, 173.1 and 171.6), which were correlated with protons of two methylenes (δ_H_ 2.55, 2.25, m, each 2H; and δ_C_ 36.7 and 36.5) of two *n*-butyrates and the methyl protons (δ_H_ 2.09 s, 3H and δ_C_ 21.4) of one acetate group, respectively. The planar structure of **5** was established by ^1^H–^1^H COSY and HMBC correlations (Figure 2). The HMBC connectivities from H-2 (δ_H_ 3.63 br s, 1H) and H-8 (δ_H_ 5.27 d, 1H, *J* = 9.2 Hz) to two carbonyl carbons δ_C_ 173.3 (C) and 173.1 (C) determined the positions of the two *n*-butyrates at C-8 and C-3. Also, the location of an acetate group at C-6 was supported by the HMBC connectivities from both of the acetate methyl protons (δ_H_ 2.09 s, 3H) and oxygenated methine proton (δ_H_ 5.71 d, 1H, *J* = 5.6 Hz) to the carbon resonating at δ_C_ 171.6 (C). The relative configuration of **5** was confirmed by analyzing the key NOE correlations (Figure 3).

**Figure 4:**
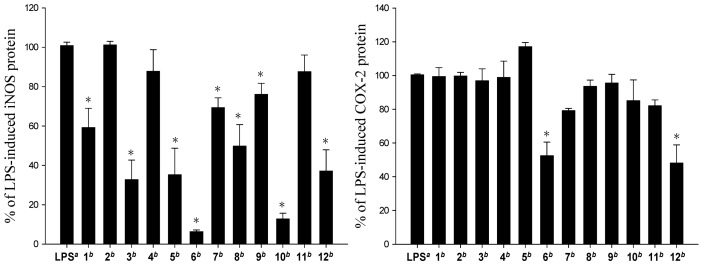
Effect of compounds **1**–**12** on LPS-induced iNOS and COX-2 proteins expression in RAW264.7 macrophage cells by immunoblot analysis. The values are mean ± SEM. (*n* = 6). Relative intensity of the LPS alone stimulated group was taken as 100%.* Significantly different from LPS alone stimulated group (* *P* < 0.05). ^a^ stimulated with LPS, ^b^ stimulated with LPS in the presence of **1**–**12** (10 μM).

The cytotoxicity of the diterpenoids **1**–**12** against the growth of five human carcinoma cells A549, BT483, H1299, HepG2, SAS and one human normal cell line BEAS2B was studied (Table 3). The results showed that **1**–**4**, **7**–**9** and **11** are not cytotoxic toward the above cancer and normal cells. Compounds **5**, **6**, **10** and **12** exhibited cytotoxicity toward the above five cancer cell lines and the human normal cell line; **10**, being the most cytotoxic. The *in vitro* anti-inflammatory effects of compounds **1**–**12** were also tested by examining the inhibitory activity of these compounds toward the LPS-induced up-regulation of pro-inflammatory proteins iNOS and COX-2, in RAW264.7 macrophage cells (Figure 4). At a concentration of 10 μM, compounds except **2**, **4**, and **11** were found to significantly reduce the expression of iNOS protein, relative to the control cells stimulated with LPS only. Among them, **6** and **10** could potently reduce the levels of iNOS protein to 6.4 ± 0.8% and 12.8 ± 2.9%, respectively. Compounds **6** and **12** also effectively reduced COX-2 expression (52.5 ± 8.0% and 48.1 ± 10.8%, respectively) in the same LPS-stimulated cells. These results revealed that *n*-butyryloxy group at C-3 could significantly enhance the cytotoxic and anti-inflammatory activities in eunicellin-type compounds. Overall, compounds **6**, **10** and **12** exhibited interesting cytotoxic and anti-inflammatory activity and could become lead compounds in the future drug development.

**Table 3:** Cytotoxicity (ED_50_ μg/mL) of compounds **5**, **6**, **10** and **12**.

	Cell Lines					Normal Cell Line
Compounds	A549	BT483	H1299	HepG2	SAS	BEAS2B
**5**	15.0 ± 3.5	11.5 ± 1.8	19.2 ± 4.0	12.9 ± 3.1	10.2 ± 3.5	– ^a^
**6**	16.1 ± 1.2	10.0 ± 1.8	11.8 ± 1.0	– ^a^	17.2 ± 0.4	10.4 ± 0.3
**10**	6.8 ± 1.0	11.6 ± 2.8	6.7 ± 0.7	8.5 ± 1.3	9.5 ± 3.7	4.8 ± 0.7
**12**	12.2 ± 1.1	6.8 ± 0.6	12.8 ± 1.2	11.1 ± 0.4	10.3 ± 0.5	13.6 ± 0.5
Taxol	1.5 ± 0.9	3.9 ± 0.8	1.2 ± 0.1	1.4 ± 0.7	2.3 ± 1.5	2.3 ± 1.5

^a^ IC_50_ > 20 μg/mL.

## 3. Experimental Section

### 3.1. General Experimental Procedures

Optical rotations were measured on a JASCO P-1020 polarimeter. IR spectra were recorded on a JASCO FT/IR-4100 infrared spectrophotometer. ESIMS were obtained with a Bruker APEX II mass spectrometer. NMR spectra were recorded either on a Varian UNITY INOVA-500 FT-NMR, a Varian 400MR FT-NMR. Silica gel (Merck, 230–400 mesh) was used for column chromatography. Precoated silica gel plates (Merck, Kieselgel 60 F-254, 0.2 mm) were used for analytical TLC. High performance liquid chromatography was performed on a Hitachi L-7100 HPLC apparatus with a ODS column (25.0 × 21.2 mm, 5 μm).

### 3.2. Animal Material

*C. krempfi* was collected by hand using scuba off the coast of Penghu islands of Taiwan in June 2008, at a depth of 5–10 m, and stored in a freezer until extraction. A voucher sample (specimen no. 200806CK) was deposited at the Department of Marine Biotechnology and Resources, National Sun Yat-sen University.

### 3.3. Extraction and Separation

The octocoral (1.1 kg fresh wt) was collected and freeze-dried. The freeze-dried material was minced and extracted exhaustively with EtOH (3 × 10 L). The EtOH extract of the frozen organism was partitioned between CH_2_Cl_2_ and H_2_O. The CH_2_Cl_2_-soluble portion (14.4 g) was subjected to column chromatography on silica gel and eluted with EtOAc in *n*-hexane (0%–100% of EtOAc, stepwise) and then further with MeOH in EtOAc with increasing polarity to yield 41 fractions. Fraction 28, eluted with *n*-hexane–EtOAc (1:2), was rechromatoraphed over a reversed-phase column (RP-18) using acetone–H_2_O (10:1) as the mobile phase to afford six subfractions (A1–A6). Subfraction A1 was repeatedly separated by reverse phase HPLC (CH_3_CN–H_2_O, 1:1 to 2:1) to afford compounds **1** (3.5 mg), **2** (4.9 mg), **3** (7.6 mg), **4** (4.8 mg), and **9** (3.6 mg). Subfraction A2 separated by reverse phase HPLC (CH_3_CN–H_2_O, 3.8: 1) to afford compounds **5** (7.1 mg), **6** (16.4 mg), **10** (30.2 mg), **11** (5.2 mg) and **12** (5.4 mg). Subfraction A3 by reverse phase HPLC (CH_3_CN–H_2_O, 1:1) to afford compound **7** (13.2 mg). Subfraction A4 by reverse phase HPLC (MeOH–H_2_O, 2.4:1) to afford compound **8** (5.5 mg). 

#### 3.3.1. Krempfielin E (**1**)

Colourless oil; [α]_D_^25^ −78.3 (*c* 0.35, CHCl_3_); IR (neat) ν_max_ 3421, 3072, 2931, 1732, 1648, 1447, 1372, 1238, 1180 and 1039 cm^−1^; ^1^H and ^13^C NMR data, see Table 1, Table 2; ESIMS *m/z* 487 [M + Na]^+^; HRESIMS *m/z* 487.2669 [M + Na]^+^ (calcd for C_26_H_40_O_7_Na, 487.2672). 

#### 3.3.2. Krempfielin F (**2**)

Colourless oil; [α]_D_^25^ −7.3 (*c* 0.49, CHCl_3_); IR (neat) ν_max_ 3073, 2959, 1736, 1645, 1454, 1369, 1240, 1165 and 1095 cm^−1^; ^1^H and ^13^C NMR data, see Table 1, Table 2; ESIMS *m/z* 443 [M + Na]^+^; HRESIMS *m/z* 443.2408 [M + Na]^+^ (calcd for C_24_H_36_O_6_Na, 443.2409). 

#### 3.3.3. Krempfielin G (**3**)

Colourless oil; [α]_D_^25^ −26.3 (*c* 0.76, CHCl_3_); IR (neat) ν_max_ 3459, 3078, 2961, 1735, 1645, 1456, 1371, 1238, 1177 and 1075 cm^−1^; ^1^H and ^13^C NMR data, see Table 1, Table 2; ESIMS *m/z* 487 [M + Na]^+^; HRESIMS *m/z* 487.2675 [M + Na]^+^ (calcd for C_26_H_40_O_7_Na, 487.2672). 

#### 3.3.4. Krempfielin H (**4**)

Colourless oil; [α]_D_^25^ −5.8 (*c* 0.48, CHCl_3_); IR (neat) ν_max_ 3077, 2958, 1734, 1645, 1455, 1369, 1240, 1165 and 1095 cm^−1^; ^1^H and ^13^C NMR data, see Table 1, Table 2; ESIMS *m/z* 443 [M + Na]^+^; HRESIMS *m/z* 443.2408 [M + Na]^+^ (calcd for C_24_H_36_O_6_Na, 443.2409). 

#### 3.3.5. Krempfielin I (**5**)

Colourless oil; [α]_D_^25^ −18.3 (*c* 0.35, CHCl_3_); IR (neat) ν_max_ 3460, 3069, 2960, 1733, 1646, 1448, 1370, 1254, 1181 and 1089 cm^−1^; ^1^H and ^13^C NMR data, see Table 1, Table 2; ESIMS *m/z* 559 [M + Na]^+^; HRESIMS *m/z* 559.3243 [M + Na]^+^ (calcd for C_30_H_48_O_8_Na, 559.3247).

### 3.4. Cytotoxicity Testing

Cell lines were purchased from the American Type Culture Collection (ATCC). Cytotoxicity assays of compounds **1**–**12** were performed using the MTT [3-(4,5-dimethylthiazol-2-yl)-2,5-diphenyl-tetra-zolium bromide] colorimetric method [25,26]. 

### 3.5. *In Vitro* Anti-Inflammatory Assay

Macrophage (RAW264.7) cell line was purchased from ATCC. In vitro anti-inflammatory activities of compounds **1**–**12** were measured by examining the inhibition of lipopolysaccharide (LPS) induced upregulation of iNOS (inducible nitric oxide synthetase) and COX-2 (cyclooxygenase-2) proteins in macrophages cells using western blotting analysis [27,28].

## 4. Conclusions

New eunicellin-based diterpenoids were isolated together with known ones from the soft coral *Cladiella krempfi.* Compounds **5**, **6**, **10** and **12** showed cytotoxicity toward the above five cancer cell lines, and one human normal cell line. Also, **6**, **10** and **12** could significantly reduce the accumulation of pro-inflammatory proteins iNOS and COX-2. Thus, these compounds, in particular **6**, **10** and **12** could be promising bioactive agents and may warrant further biomedical investigation.
